# Genome wide association studies are enriched for interacting genes

**DOI:** 10.21203/rs.3.rs-5189487/v2

**Published:** 2024-10-22

**Authors:** Peter T. Nguyen, Simon G. Coetzee, Irina Silacheva, Dennis J. Hazelett

**Affiliations:** Cedars-Sinai Medical Center; Cedars-Sinai Medical Center; Cedars-Sinai Medical Center; Cedars-Sinai Medical Center

**Keywords:** GWAS, genetic algorithms, variant prioritization, multi-omics, breast cancer, complex disease, etiology, susceptibility, gene network

## Abstract

**Background:**

With recent advances in single cell technology, high-throughput methods provide unique insight into disease mechanisms and more importantly, cell type origin. Here, we used multi-omics data to understand how genetic variants from genome-wide association studies influence development of disease. We show in principle how to use genetic algorithms with normal, matching pairs of single-nucleus RNA- and ATAC-seq, genome annotations, and protein-protein interaction data to describe the genes and cell types collectively and their contribution to increased risk.

**Results:**

We used genetic algorithms to measure fitness of gene-cell set proposals against a series of objective functions that capture data and annotations. The highest information objective function captured protein-protein interactions. We observed significantly greater fitness scores and subgraph sizes in foreground *vs.*matching sets of control variants. Furthermore, our model reliably identified known targets and ligand-receptor pairs, consistent with prior studies.

**Conclusions:**

Our findings suggested that application of genetic algorithms to association studies can generate a coherent cellular model of risk from a set of susceptibility variants. Further, we showed, using breast cancer as an example, that such variants have a greater number of physical interactions than expected due to chance.

## Background

The primary goal of genome-wide association studies (GWAS) is to catalog and translate genetic variants to uncover disease mechanisms [1–3]. Over the past twenty years, researchers leveraged GWAS to pinpoint specific genomic regions for further investigation [4, 5]. However, one of the challenges of interpreting GWAS is that 95% of single nucleotide polymorphisms (SNPs) fall outside of the protein coding region [6–8]. Depending on the linkage disequilibrium (LD) structure, anywhere from one to hundreds of non-functional SNPs may be associated with a disease at a single locus [9]. Thus, identification of the causal variant and gene poses great difficulty.

Considerable work has gone into analyzing and interpreting GWAS data [2, 4–6, 9–12]. *FunciSNP* [6] and *HaploReg* [13] were developed to identify candidate functional SNPs in non-coding regions by integrating biofeatures such as SNPs with high LD, epigenomic data, and DNA-binding factors. The impact of functional SNPs has been tested through *in vitro* multi-tissue expression quantitative trait loci (eQTL) to find gene associations [10].

More recently, machine learning approaches through aggregation of multi-omics data were developed to improve prioritization [8, 11]. Mountjoy *et al.* developed a locus-to-gene (L2G) pipeline that integrates QTL, gene distance, and pathogenicity predictions to rank likely causal genes [11]. While their method provides statistical evidence for prioritization, they don’t account for cell type specificity [11]. The use of single-cell sequencing technology provided unique insights into molecular mechanisms. Corces *et al.* used bulk and single-cell assay for transposase-accessible chromatin sequencing (ATAC-seq) data to identify cell type specific open chromatin to prioritize gene and cell type of noncoding GWAS loci in neurodegenerative diseases [8]. Zhang *et al*. developed single-cell disease relevance score (scDRS), which exploits single-cell RNA sequencing (scRNA-seq) data and associates disease specific expression signatures with specific cell populations [14].

One of our prior studies connected SNPs to genes encoding both ligands and their cognate receptors [12]. The existence of ligand receptor pairs in GWAS implies intercellular communication as part of susceptibility – highlighting the potential role of other cell types besides the cell-of-origin [12]. What is currently lacking from attempts to integrate single-cell omics and GWAS data is that multiple independent genetic signals may produce similar cellular effects through protein interaction networks.

Our hypothesis is that variants associated with cancer affect interacting proteins and cell types to promote disease initiation. Based on this hypothesis, we predict that accounting for physical interaction of susceptibility genes will increase sensitivity and accuracy. Here, we use genetic algorithms (GA) to integrate breast cancer (BCa) GWAS with interaction data, single-nucleus RNA-seq (snRNA-seq) data, single-nucleus ATAC-seq (snATAC-seq) data, and genome annotations to prioritize gene and cell type at each locus.

## Methods

### GWAS data.

We obtained BCa variants from NHGRI-EBI GWAS Catalog [15, 16]. That data was derived from cases and controls of European ancestry from studies using the Breast Cancer Association Consortium (BCAC) [17] and Consortium of Investigators of Modifiers of BRCA1/2 (CIMBA) [18] (Table 1). We identified the most recent BCa GWAS [16] that expanded on previous BCAC GWAS [19–21]. We performed LD expansion using *LDlinkR* [22] for the European population. Due to genetic drift, the Finnish population was excluded. We selected proxy SNPs with MAF ≥ 5%, R^2^ ≥ 0.6, D′ ≥ 0.9.

### Single-nuclei data.

**Figure 1:**
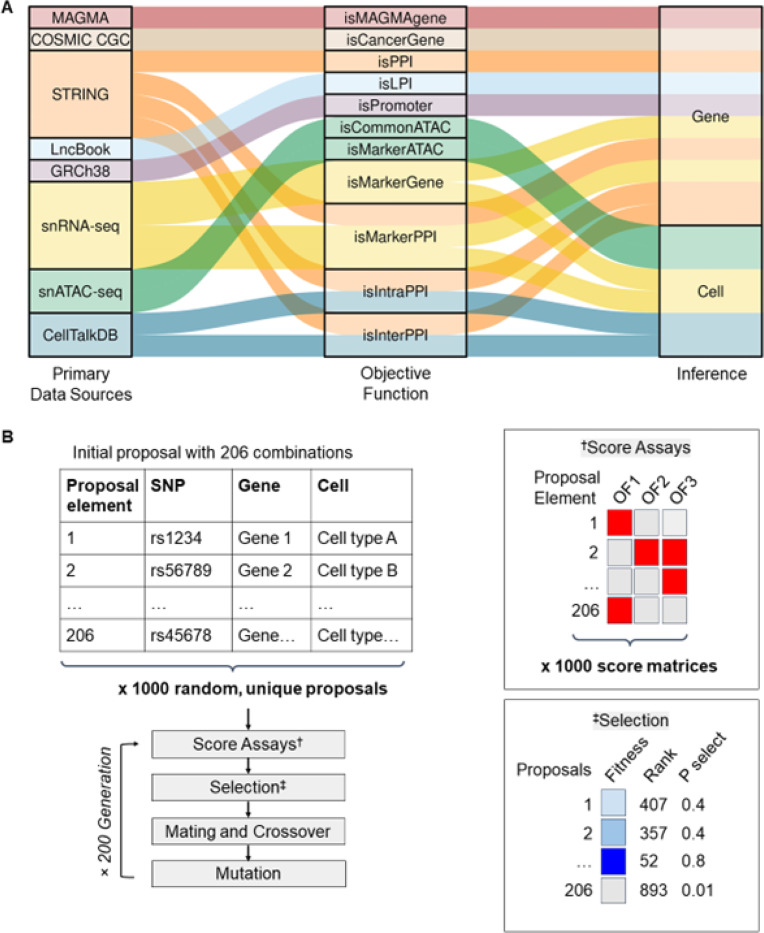
Integration of GWAS, genome annotation, and interaction data using genetic algorithm. (A) The relationships of data sources to individual objective functions and gene *vs.*cell type inferences. (B) Schematic of GA method used in this study.

We identified published data with normal breast tissue, including matching pairs of samples for snRNA-seq (GSE168836) and snATAC-seq (GSE168837) [23] (Table 1, Fig. 1A). We applied *sctransform* [24] for cell-to-cell normalization and variance stabilization on the RNA dataset and used provided scripts (process_atac.R) from [23] to acquire the peak matrix by cell type.

### Identification of candidate genes.

We used protein coding genes and lncRNAs from the 10X Genomics human reference “*refdata-gex-GRCh38–2020-A*” [23]. To identify candidate genes (“nearby gene set”), we defined a window size using the minimum and maximum chromosome positions from the lead SNP and its proxies. We expanded the window by 200 kb on each side to account for adjacent genes and imposed a minimum of five genes up- and downstream for each lead SNP.

### Genetic algorithm.

The GA model consists of five steps: 1) generate a population of 1,000 random proposals (potential solution); 2) score proposal “fitness” as the average of all objective functions (OFs); 3) select pairs of proposals for mating with probability proportional to fitness rank; 4) introduce mutations for gene and cell type; 5) repeat steps 2–4 for 200 generations (Fig. 1B).

### Initiation of proposals.

The number of proposal elements (gene-cell type combinations) is equal to the number of lead SNPs. Each element consists of a lead SNP, a gene, and a cell type. We randomly select a gene from nearby gene sets and cell type using the cell labels from the snRNA- and snATAC-seq data [23].

### Building objective functions (OFs).

We created OFs (names italicized throughout the text) using external data sources for gene and cell type prioritization (Fig. 1A). For gene prioritization, these functions are: *isMAGMAgene*, *isCancerGene*, *protein-protein interaction* (*isPPI*), *lncRNA and protein interaction* (*isLPI*), and *isPromoter*. For cell type prioritization, these OFs capture: non-cell type specific ATAC peaks - *isCommonATAC and* and cell type specific peaks - *isMarkerATAC.* For data that inform on both gene and cell type, these functions are: *isMarkerGene*, *isMarkerPPI*, *intracellular PPI* (*isIntraPPI)* and *intercellular PPI* (*isInterPPI*). These datasets and their relationships to the OFs are described in Table 1 and Fig. 1A.

### Conversion of breast data sources to boolean values in OFs.

All OFs were scored as boolean values on each proposal element. For *isMAGMAgene*, we obtained a set of genes from Multi-marker Analysis of Genomic Annotation (MAGMA) database [25] based on the 2013 UK biobank 460k release for BCa, which uses GWAS summary statistics to identify genes strongly associated with the phenotype [14]. We scored loci as positive when the proposed gene is from the MAGMA gene set. For *isCancerGene*, we combined BCa associated gene mutations and gene fusions from COSMIC Cancer Gene Census [26] to curate the cancer gene set. We scored based on membership in this set, similar to *isMAGMAgene*. For *isPPI*, we selected protein-protein interactions with experimental evidence > 0 from STRING v11.5 [27]. We scored *isPPI* by identifying proposed genes at different loci found in STRING. We scored *isLPI* by identifying proposed genes at different loci found in LncBook [28]. In *isPromoter*, we identified variants in promoter regions (defined as 1 kb upstream and 100 bp downstream of transcription start site (TSS)) using the 10X Genomics human reference genome. We scored loci that reside in a promoter region of the proposed gene. In *isCommonATAC*, we used the peak matrix described in “GWAS and Single-nuclei data.” Since enhancers bind regulatory factors in the regions immediately flanking open chromatin, we identified peaks that contain SNPs with cell type annotation. SNPs in ATAC-seq peaks found in multiple cell types were labeled as “common.” In *isMarkerATAC*, we identified cell type specific peaks [29] at FDR ≤ 0.05 and log_2_ fold change ≥ 0.25. In *isMarkerGene*, we used the count matrix data, as previously described in “GWAS and Single-nuclei data,” to identify gene expression markers for each cell type [30] using a different cell type as the background. We selected genes at p ≤ 0.05 and log_2_ fold change ≥ 0.25. In *isMarkerPPI*, a combination of *isPPI* and *isMarkerGene*, we scored as positive when two conditions were met: 1) both proposed genes participate in PPI, 2) the proposed cell type is a valid cell type marker. In *isInterPPI*, we filtered for genes found in CellTalkDB [31], a database of ligand-receptor interactions. We scored as positive any two loci with proposed genes in a ligand-receptor interaction and the cell types are heterogeneous. In contrast, we removed CellTalkDB genes to curate *isIntraPPI*. For *isIntraPPI*, we scored similarly to *isInterPPI*, but the proposed cell type must be the same.

### Selection.

To identify parent proposals for the next generation, we use a fitness rank proportional selection method. To accomplish this, we compute fitness score as the arithmetic mean of all OFs. We rank proposals from highest to lowest and divide them into five equal groups (Group 1 being the highest rank). We sample 100 proposals without replacement using group probability for mating and crossover. We replace a Group 5 proposal at random with the top proposal (“elite”) in the current generation. During crossover, we select 50% proposal elements (loci) at random from the first proposal, then select the complementary half from the second proposal. We combine the results to construct a child proposal. For each of the 100 parent proposal pairs, we generate 10 child proposals for the next generation, for a total of 1,000.

### Mutation.

We implemented a 1% mutation rate on gene and cell type for each child proposal. For gene mutation, we randomly selected a gene from the nearby gene set to replace the current gene. For cell type, we similarly selected a random cell label to replace the current one.

### Termination of the algorithm.

**Figure 2:**
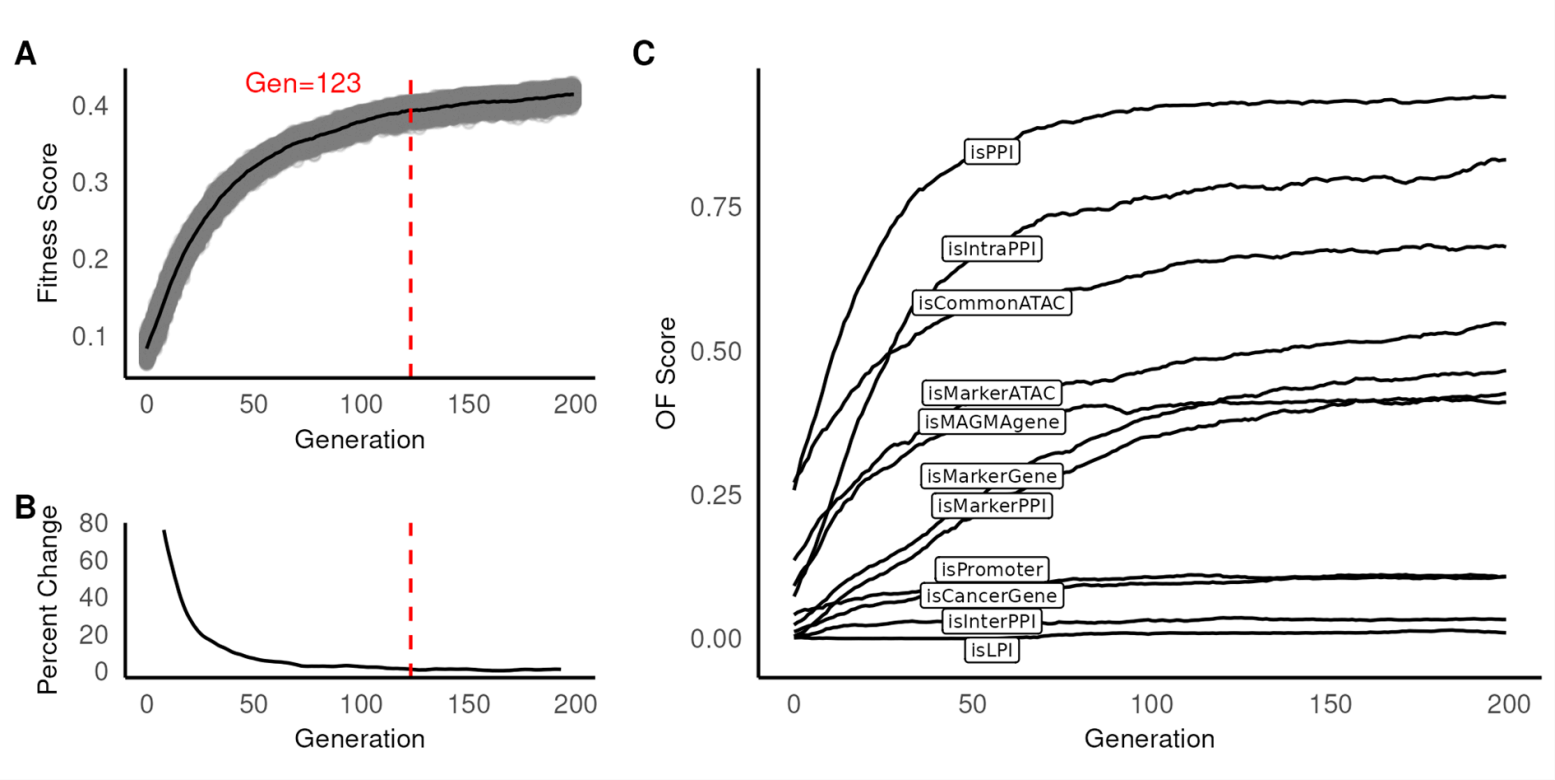
BCa GWAS data with GA. (A) Trace plot of fitness score for all generations. Vertical axis shows fitness score, horizontal is the generation. Each dot represents the fitness for a proposal. Black solid line represents the average fitness score for each generation. Red dashed line represents generation when fitness score begins to stabilize (reached optimal solution). (B) Trace plot of fitness score percent change smoothed over five generations. Vertical axis shows fitness score percent change, horizontal is the generation. Red dashed line represents the generation when ≤ 1% change in fitness score is observed for the next 10 generations. (C) Trace plot of individual OF scores. Vertical axis shows fitness score, horizontal is the generation.

We repeated steps 2–4 until the fitness score variance < 1% for 10 generations (Fig. 2A, B), empirically determined to be 123 generations. We rounded this number up to 200 for all subsequent trials.

### Curation of control SNPs.

We used *vSampler v1.2.1* [32] to generate control variants matching BCa SNPs (Supplemental Fig. 1). We used the following parameters: MAF (± 0.05), distance to closest TSS (± 100 kb), gene density (± 20 in ± 200 kb), number of proxy SNPs in LD (± 75 for R^2^ > 0.8), and enabled sampling across chromosome (Supplemental Fig. 2A). For computed parameters, we selected a value two standard deviations away from the mean. Using the GWAS lead SNPs as a model, we identified 10 matched control variants for each locus. We randomly selected 10 matching sets, each set mirrors 176 of the 206 BCa variants. Thirty SNPs were excluded as insufficiently matching our criteria. We observed low similarity between the candidate gene lists in the control sets and BCa GWAS (Supplemental Fig. 2B). We chose this many controls to estimate the variation or noise inherent in a set of variants of equal size.

### OF enrichment calculation.

We calculated enrichment of an OF in BCa as the posterior probability of observing the fraction of positives in the OF compared to control. We defined enrichment exclusion of zero from the 95% range of credible differences.

## Results

### Optimization of gene-cell proposals against breast data using GA

To describe the mechanisms of cancer risk based on population genetics of BCa, we acquired 206 lead variants of European ancestry [16, 19–21] (Table 1). For each variant, we identified proxy SNPs in LD plus candidate genes (described in Methods). These SNPs were within 200 kb of 2,292 genes of which 51% (n = 1,175) were protein-coding. To better understand these SNPs in the context of normal breast, we identified matching pairs of samples for snRNA-seq and snATAC-seq [23] (Table 1, Fig. 1A). Within these data, cells were divided into 10 clusters: hormone receptor-positive and -negative luminal cells, basal cells, blood and lymphatic endothelial cells, vascular accessory cells, adipocytes, fibroblasts, myeloid, and lymphoid cells.

The biggest challenge is the large number of combinations of hypotheses for every locus. In this study, there are at least 10^206^ combinations of plausible solutions when considering only genes. We chose GA to identify the most plausible gene and cell set (“proposal”) based on diverse evidence sources. The evidence sources for gene and cell type prioritization are captured in a set of named objective functions described in the Methods and Table 1.

We optimized for 200 generations and then analyzed the proposals in the last generation (Gen200) to assess the result (Fig. 2A, B). We observed that information was distributed unevenly between OFs: the mean score for *isCancerGene*, *isLPI, isPromoter*, and *isInterPPI* were less than 0.1 (Fig. 2C), whereas *isPPI* had the highest score (0.941). The remaining OFs had scores ranging from 0.410 to 0.832. When compared to other proposals, the elite proposal did not have top scores in all OFs. We asked whether consensus solutions might have a higher score than the elite proposal. To do this, we identified the top gene and cell type for all loci across 1,000 Gen200 proposals. Surprisingly, we observed a fitness score of 0.433 for the consensus – an improvement over the elite proposal (0.429). We observed no change for *isMAGMAgene*, *isCancerGene*, *isPPI*, and *isPromoter* between the consensus and elite proposal. However, we did observe higher OF scores for *isMarkerGene*, *isMarkerPPI*, *isIntraPPI*, *isInterPPI, isCommonATAC* and *isMarkerATAC*, and lower OF scores for *isLPI* in the consensus compared to the elite proposal. This result suggests the existence of multiple, mutually exclusive, but equally stable solutions preserved only in the consensus proposal.

### GA identifies known targets

We compared genes discovered in the consensus against L2G [11] and a naive nearest gene classifier (distance from TSS). L2G outputs the likelihood a gene is causal for the SNP (L2G score) based on distance, molecular QTL, chromatin interaction and variant pathogenicity. We identified the same SNPs across the dataset and selected the gene with the highest L2G score. Of the 175 common loci, we observed 46.8% (n = 82) with shared prediction between L2G and consensus. Across all three models, 68 loci shared the same gene. In total, 77.7% (136 out of 175 loci) L2G genes were the nearest gene to the SNP, so we did not expect our model to have high concordance with L2G because we did not include a gene distance OF. While gene distance to SNP is worth consideration, it has been reported that the nearest gene to the SNP is affected only 15% of the time [33]. In contrast, in our predictions, 41.7% (86 out of 206 loci) were the nearest gene, an intermediate value between these two figures.

**Figure 3:**
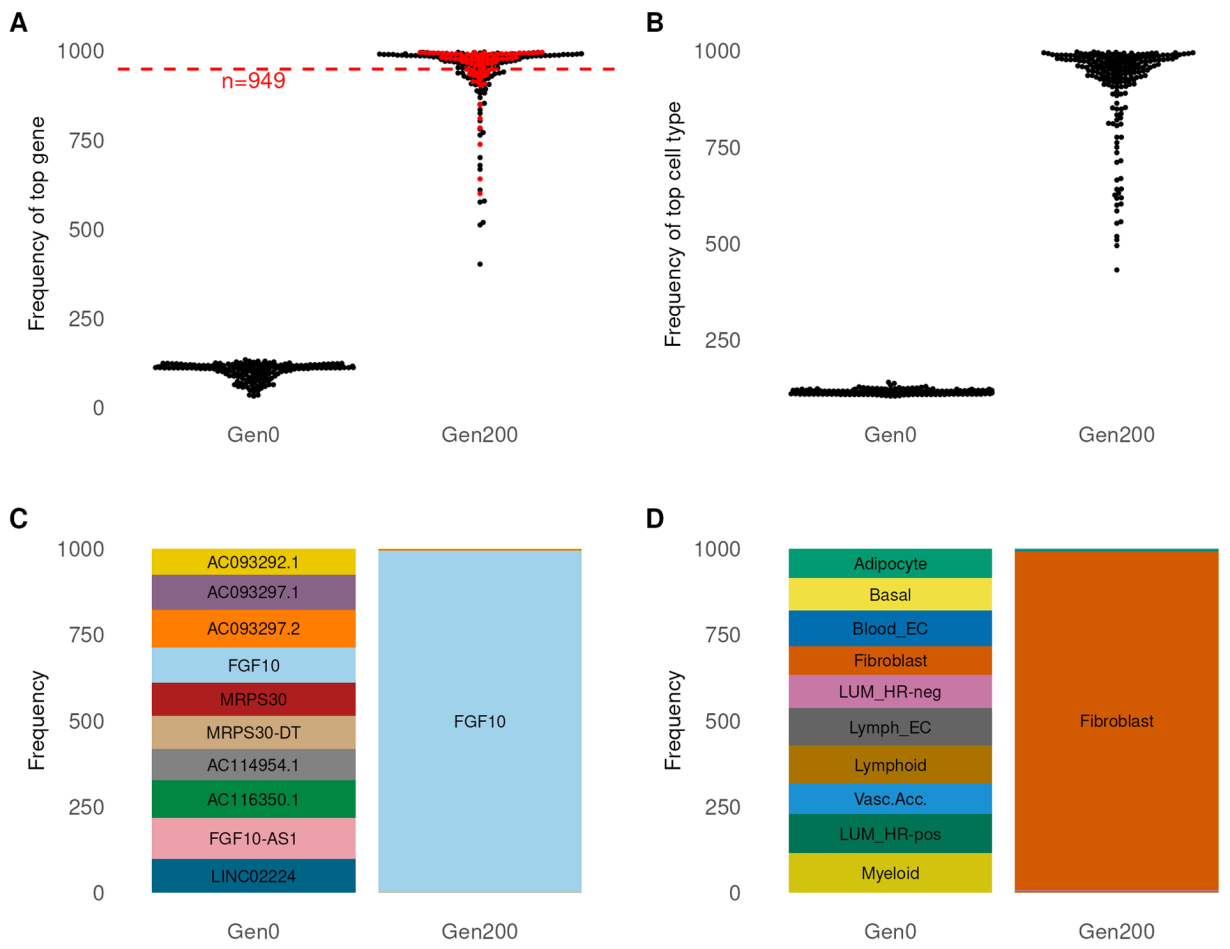
Identification of high confidence gene and cell type. (A) and (B) Distribution of proposal frequencies for top genes (A) and cell types (B) called at each locus across all proposals in Gen0 and Gen200. Each dot represents a locus. Red dot represents L2G loci with similar gene prediction to consensus solution. (C) and (D) Distribution of proposed gene (C) and cell type (D) for rs10941679 across all proposals in the Gen0 and Gen200.

The identification of high confidence gene and cell type calls are essential for downstream analysis. We performed a power calculation to determine the threshold for identifying high confidence calls. To do this, we selected a threshold where 80% of high confidence L2G SNPs with the same gene prediction as the consensus (L2G ≥ 0.7) are detected (949 proposals) (Fig. 3A). We used this same threshold to identify high confidence cell types (Fig. 3B). The number of loci with a high confidence call in gene and cell type are 147 and 118 out of 206 respectively. At lead SNP rs10941679, we found the top gene and cell type was *FGF10* and “fibroblast” in Gen200 (Fig. 3C, D). Compared to L2G, *MRPS30* (L2G = 0.542) was ranked higher than *FGF10* (L2G = 0.145) for the same SNP due to support from the QTL and distance modules [11]. Interestingly, eQTL analysis with rs10941679 revealed changes in gene expression levels for *MRPS30* and *FGF10* in MCF7 and BT474 BCa cell lines [34]. In our model, we observed shared evidence (*isMAGMAgene* and *isPPI*) for both genes. However, *FGF10* had *isMarkerGene* as additional evidence. This result highlights the ability of our model to account for complex interactions and mechanisms.

### Contribution of individual OFs to overall fitness

We assessed each OF’s contribution to fitness by comparing information content between Gen0 and Gen200. To do this, we computed the posterior probability of observing an OF score in Gen200 given Gen0. We also computed the effect size (ES) as the median difference between the two distributions. We found that the most informative OFs were *isPPI* (ES = 0.744) and *isIntraPPI* (ES = 0.870). We expected *isMAGMAgene*, which captures gene expression as a function of GWAS, to be the most informative OF. Although informative, *isMAGMAgene* yielded a lower score (ES = 0.359) than the top OF. In contrast, *isCancerGene* was not informative (ES = 0.106). *IsLPI* was also not informative (ES = 0.008), possibly due to a low number of lncRNA in the consensus (n = 5). For cell type prioritization, we observed *isCommonATAC* (ES = 0.438) and *isMarkerATAC* (ES = 0.398) to be informative, as expected.

We next investigated the information content on a locus-by-locus basis. To accomplish this, we counted all loci with OF support in Gen0 and Gen200. We used Kolmogorov-Smirnov (KS) to test whether these observations derive from the same theoretical distribution (KS test p = 2.20 × 10^− 16^). In Gen0, we observed 54.8% loci (n = 113) without OF support. In contrast, every locus had at least one supporting OF in Gen200. Additionally, Gen200 had more supporting OFs per locus (μ = 4.76, SD = 1.64) when compared to Gen0 (μ = 0.8, SD = 1.04). Taken together, five OFs had evidence for a large number of positive loci (*isMAGMAgene, isPPI*, *isCommonATAC*, *isMarkerATAC*, and *isIntraPPI*).

### BCa GWAS loci are enriched in associations with breast-specific assays

We reasoned that, according to our hypothesis, in which GWAS variants interact to link common cell types and pathways, there should be a greater number of associations both with disease relevant data and between loci. To test these predictions, we first evaluated whether the solutions discovered by GA had higher fitness than those from equivalent sets of randomly selected variants. Second, we analyzed the network properties of BCa GWAS relative to these control sets.

**Figure 4:**
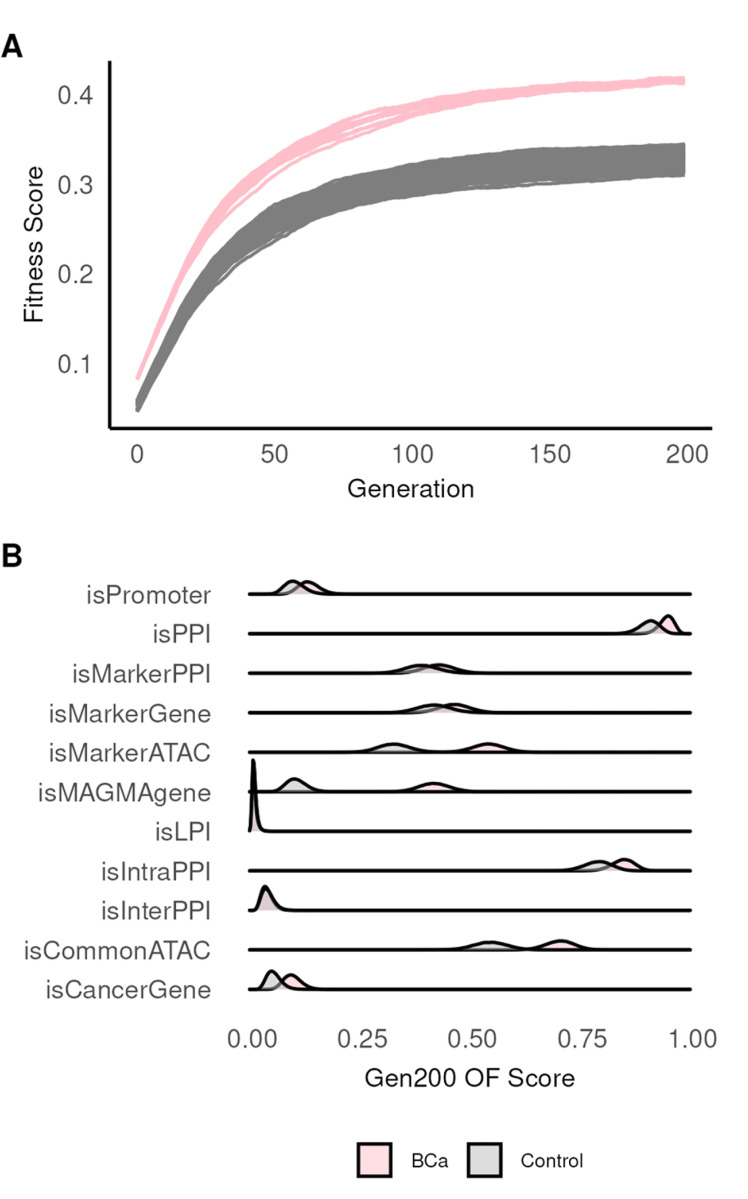
Comparison of fitness and OF scores in BCa and control set. (A) Trace plot of fitness score for 10 BCa and 100 control runs. Vertical axis shows fitness score, horizontal is the generation. (B) Distribution of individual OF scores in the Gen200 for BCa and control. Vertical axis is OF, horizontal shows fraction of positives (out of maximum loci).

We repeated GA as before with our 10 control sets. To capture random variation in stable solutions we ran nine additional models for BCa and each control set, each with a different initial population, (10 BCa and 100 control GA runs) (Fig. 4A). In Gen200, we computed the posterior probability of observing the BCa fitness scores given the control distribution (BCa: μ = 0.415, SD = 1.83 × 10^− 3^; control: μ = 0.330, SD = 8.77 × 10^− 3^). We observed the BCa fitness scores were significantly higher compared to the control by assessing the probability that the mean difference is zero or less (p = 0.041). Thus, our model is able to distinguish between BCa and randomly chosen SNPs. Moreover, the higher fitness score reveals the potential for true biological associations between BCa GWAS and breast derived multi-omics data.

If a higher fitness score in BCa is driven by its associations with breast-specific data, we predict that the BCa and control set fitness scores should also be driven by different OFs. To test this prediction, we computed the posterior probability of observing positive OFs in BCa given the control set (Fig. 4B). We observed *isMAGMAgene*, *isCommonATAC*, and *isMarkerATAC* higher in BCa than control (ES greater than zero, p < 0.05). We expected *isMAGMAgene* to outperform in BCa compared to the control group (ES = 0.314) as it’s derived from breast expression. The enrichment of *isCommonATAC* and *isMarkerATAC* relative to control suggests that BCa SNPs are associated with normal breast cell types. In contrast, *isPromoter, isLPI, isMarkerGene*, *isMarkerPPI*, and *isInterPPI* were indistinguishable between the BCa and control set when assessing the frequency of ES greater than zero (p ≥ 0.05) (Fig. 4B). Surprisingly, we observed *isIntraPPI* (p = 0.073) and *isPPI* (p = 0.074) had a small ES when comparing the BCa to the control set, 0.057 and 0.039 respectively. The result shows that even randomly selected SNPs have a high PPI score.

Given the enriched OFs in BCa, we asked how individual loci contributed to increased fitness over control. To address this, we measured the information content at all BCa loci. We computed the number of OF support for the 176 BCa loci used to match the control sets. We identified the consensus gene and cell type in Gen200 for the 10 BCa GA runs and the 10 matching SNPs from the 10 control GA runs (total of 10 × 10 = 100 runs) and computed the number OF support for each of the 110 GA runs. We used the Wilcoxon rank-sum test to identify differences between OF support by comparing the two distributions. After multiple hypothesis correction, we observed 61.4% (n = 108) BCa loci with higher OF support than control (p ≤ 0.05, ES > 0). In contrast, we observed 8.5% (n = 15) BCa loci with lower OF support than control (p ≤ 0.05, ES < 0). Our analysis of the result demonstrates a majority of loci in BCa have higher OF support than due to chance alone, and provides critical information about lack of support for other loci. This procedure can be used to measure the benefit of OFs, and to exclude non-informative loci from downstream analysis.

By curating a set of control SNPs, we identified the most informative OFs (*isMAGMAgene*, *isCommonATAC*, and *isMarkerATAC*) that distinguish BCa from control. These OFs corresponded to the breast specific data. We anticipated that *isPPI* would be an informative OF, but despite its overall importance to the outcome for BCa and control (OF mean = 0.94 *vs.* 0.90 controls) our analysis revealed no significant difference. It is possible that including all interaction experimental evidence (interaction score > 0) from STRING in our *isPPI* OF may not be stringent enough. It is also possible the quality of interactions as measured in network size is better in BCa than control, and we explore this next.

### GWAS variants are enriched for larger networks

**Figure 5:**
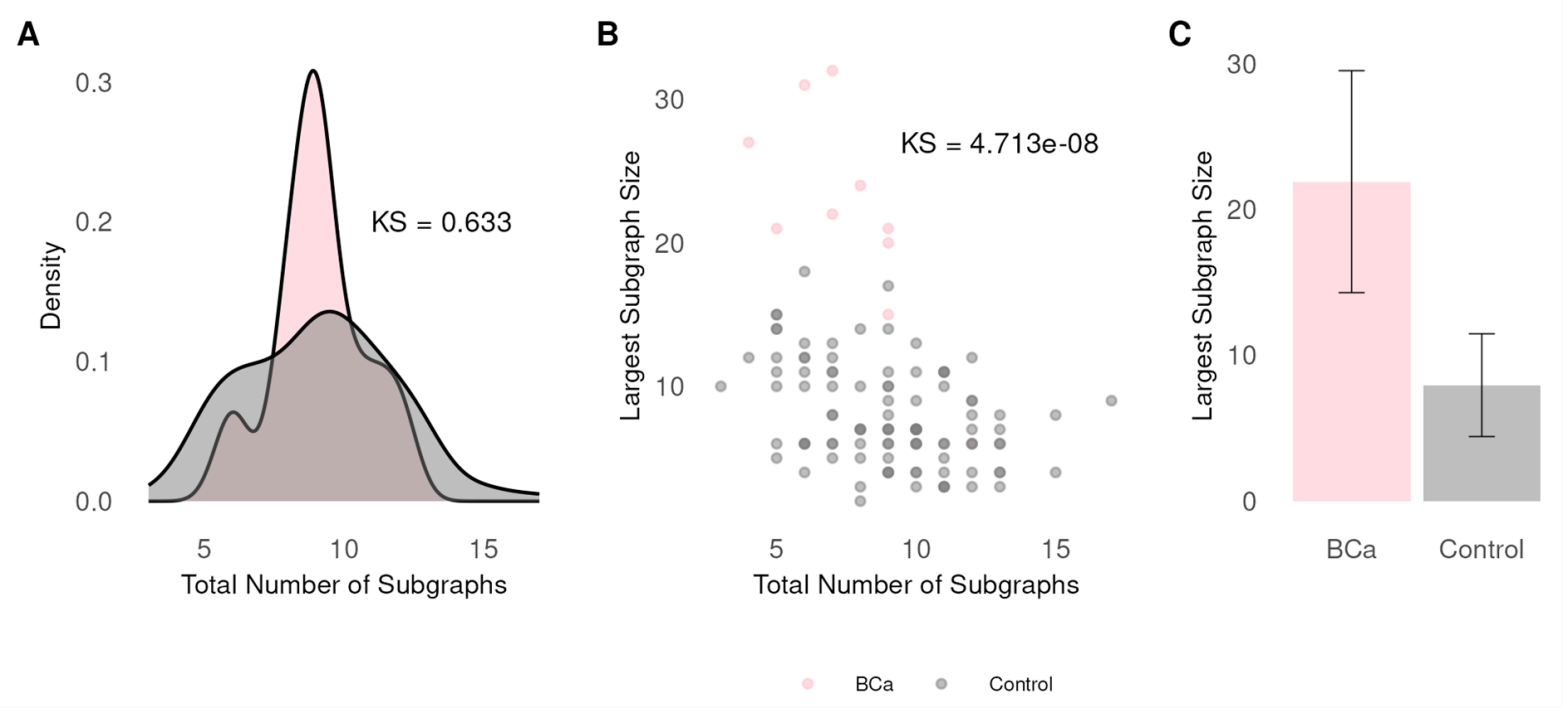
Comparison of protein-protein interaction in BCa and control set. (A) Distribution of the number of PPI subgraphs in BCa and control. Vertical axis shows density, horizontal shows the number of subgraphs. (B) Distribution of the largest PPI subgraph size in BCa and control. Each dot represents an optimized GA run (10 breast and 100 control). Vertical axis shows subgraph size, horizontal shows the number of subgraphs. (C) Gene size in largest subgraph for BCa and control. Data are presented as mean. Error bars are presented as standard deviation.

Based on our OF enrichment analysis (Fig. 4B), *PPI* failed to distinguish between the BCa and control set. This finding did not support our hypothesis that molecular interaction mechanisms are embedded within GWAS. If the control set represents variants without any true associations to breast data, then we predict BCa will have larger PPI network sizes. To test our prediction, we identified all PPI (interaction score ≥ 0.4) for the 10 BCa and 100 control GA run. We observed no significant difference in the number of subgraphs between the two groups (KS test p = 0.633) (Fig. 5A). Next, we computed the number of genes per subgraph and observed the control having fewer genes in their largest subgraph (μ = 7.95, SD = 3.52) when compared to the BCa sets (μ = 28.6, SD = 3.5). We used KS to test whether these observations derive from the same theoretical distribution (KS test p = 2.132 × 10^− 14^). Additionally, we downsampled the BCa (n = 176) to adjust for the additional 30 SNPs that we excluded in making the control sets. We observed BCa (μ = 21.9, SD = 7.61) still had more genes in their largest subgraph compared to controls (KS = 4.71 × 10^− 8^) (Fig. 5B, C). The result strongly supports the conclusion that genes selected in BCa GWAS have a larger PPI network than expected due to chance, consistent with our hypothesis that GWAS variants are functionally connected.

### Reconstruction of cellular interaction from the consensus proposal

**Figure 6:**
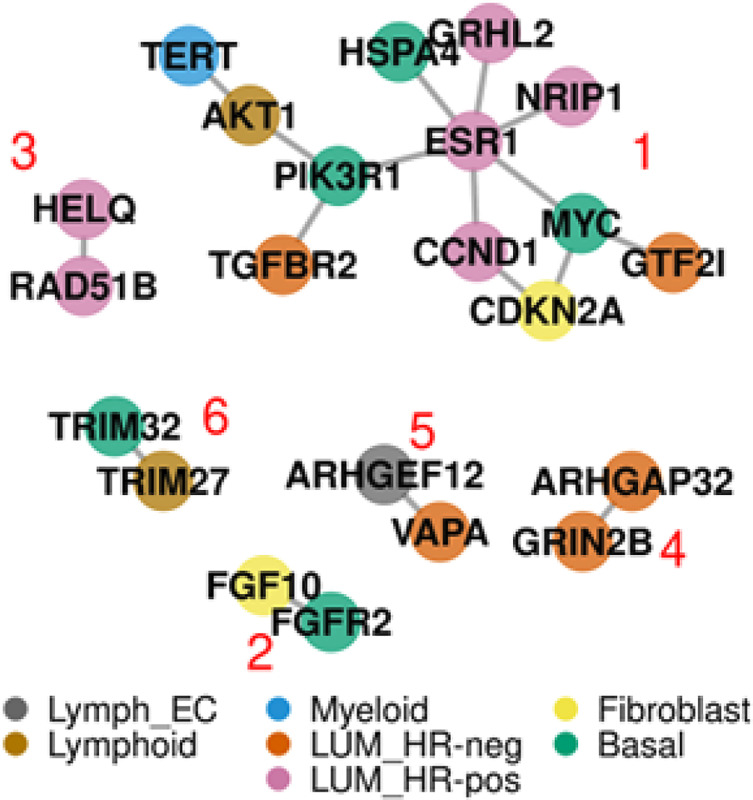
Graphical representation of PPI network for high confidence calls for genes in BCa. The genes represent high confidence calls at loci across 10 independent BCa runs, indicated by low Gini-Simpson index (low diversity). Red numbers designate subgraph membership. Node color represents cell type.

Earlier, we found the surprising result that the consensus proposal scored higher than the highest scoring elite proposal. We speculated that competing subsets of loci in different proposals produce more than one family of stable solutions. To quantify diversity of the Gen200 proposal set, we computed the Gini-Simpson index for the 206 loci in the 10 BCa GA runs. We selected loci with low diversity (Gini-Simpson index ≤ 0.5 and gene count ≤ 2) that produced the same gene predictions across multiple independent runs. Of the 118 high confidence BCa SNPs, we identified 26 loci with PPI. We constructed a projection of the protein interaction network which consisted of 6 subgraphs – the largest having a total of 12 genes (subgraph 1) (Fig. 6).

We constructed a map that links genetic variants to gene and cell type. To accomplish this, we annotated predicted cell type on the PPI network graph from Fig. 6. The largest subgraph included basal, luminal hormone receptor positive and negative, fibroblast, adipocytes and blood endothelial, and lymphatic cell types. This result shows in principle how an interpretable model of GWAS can be constructed from the consensus proposal.

## Discussion

We introduced a framework that leverages single-nucleus multi-omics, genome annotations and interaction data to prioritize gene and cell type for GWAS loci. As proof of principle, we selected BCa for study because of availability of public data, in particular matching single-nucleus multi-omics for normal breast. Our method considered all BCa GWAS loci as a single proposal rather than individually. We employed GA to evaluate, score, and modify proposals based on OFs that capture mechanisms such as disruption of promoters, open chromatin, and PPI. We applied this method to BCa and recovered known target genes. We showed BCa loci were enriched in association with OF in BCa multi-omic data and PPI when compared to equivalent sets of randomly selected variants. These analyses provided support for our hypothesis that interactions between proteins encoded at GWAS loci are an important feature of genetic association studies.

We note several limitations of our work. First, we grouped lead SNPs with proxies under the lead SNP term. In our model, the GA could use OF support from more than one SNP under the lead SNP term – masking the causal SNP. This limitation could be addressed by allowing the GA to fit data to proxy SNPs as a third parameter the way we fit nearby genes and cell type in this study.

Second, our model utilized 11 OFs. As we noted at the end, our largest subgraph was not as coherent with respect to cell type. Although it is beyond the scope of this work, we have reason to believe that future inclusion of transcription factor networks will greatly enhance the overall coherence of our models. Nonetheless, the results presented here represent the best explanation of BCa risk given the data we used. We expect the solutions to evolve as additional data and OFs are introduced.

Third, the model does not account for the independent risk of histological subtypes. As discussed above, this may contribute to competing optimizations within each proposal set. We plan to address these shortcomings in future analyses.

## Conclusions

These findings suggest that our framework is able to uncover molecular mechanisms embedded in GWAS. Future studies using GA or other artificial intelligence approaches explicitly modeling molecular interactions between loci have great potential to provide novel insight for GWAS in mediating risk.

## Supplementary Material

Supplement 1

## Figures and Tables

**Table 1:** Data sets used in this study.

Data Set	Source	Description
BCa GWAS (integrated references)	Zhang *et al*., 2020 [16], Michailidou *et al.*, 2017 [19], Garcia-Closas *et al.*, 2013[20], Milne *et al.*, 2017 [21]	The following study accessions were used: GCST001930, GCST010098, GCST010099, GCST010100, GCST004988, GCST005076, GCST005077, and GCST005075.
snRNA-seq	Raths *et al*., 2023 [23]	66,926 nuclei from 9 cis-gender females (GSE168836).
snATAC-seq	Raths *et al*., 2023 [23]	27,459 nuclei from 9 cis-gender females (GSE168837).
Marker gene		This study, reanalysis of gene expression marker set at cell type resolution from Raths *et al.*, 2023 [23].
MAGMA gene	Zhang *et al*., 2022 [14]	MAGMA, a set of putative disease genes from the 2013 UK biobank 460k release for BCa, consisting of 1,000 genes.
Cancer gene	Sondka *et al.*, 2018 [26]	COSMIC cancer gene consensus is a collection of gene mutation and fusion implicated in cancer.
Protein-protein interactions	Szklarczyk *et al.*, 2023 [27]	STRING (string-db.org), is a database of protein-protein interaction with experimental evidence.
LncRNA and protein interactions	Li *et al*., 2023 [28]	LncBook is a comprehensive resource of human lncRNA-protein interaction.
Promoter regions	10X Genomics	Promoter region extracted from prebuilt *GRCh38 genome reference version 2020-A* [23].
Common/marker ATAC peaks set		This study, reanalysis of open chromatin peak set at cell type resolution from Raths *et al*., 2023 [23].

## Data Availability

The data supporting the conclusions of this article are available in the Zenodo repository under https://zenodo.org/records/13851449. All code for producing the analyses and figures herein are included in this fully reproducible manuscript in R markdown format. R markdown files are available from our repository on the distributed version control site, Github: https://github.com/Junkdnalab/Inherited_Risk_GA. Further information and requests for resources and analyses should be directed to and will be fulfilled by the lead contact, Dennis J. Hazelett, Ph.D. (Dennis.Hazelett at csmc dot edu)

## Abbreviations

BCa: Breast cancer
BCAC: Breast Cancer Association Consortium
Bp: Base pairs
CIMBA: Consortium of Investigators of Modifiers of BRCA1/2
COSMIC: The Catalog Of Somatic Mutations In human Cancer
ES: Effect Size
eQTL: Expression quantitative trait loci
GA: Genetic algorithm
Gen0: Initial population
Gen200: Final population
GWAS: Genome-wide association studies
Kb: Kilobases
KS: Kolmogorov-Smirnov
L2G: Locus-to-gene
LD: Linkage disequilibrium
LPI: LncRNA-protein interaction
MAGMA: Multi-marker Analysis of Genomic Annotation
MAF: Minor allele frequency
OF: Objective function
PPI: Protein-protein interaction
snATAC-seq: Single-nucleus RNA sequencing
snRNA-seq: Single-nucleus assay for transposase-accessible chromatin sequencing
SD: Standard deviation
SNP: Single nucleotide polymorphism
STRING: Search Tool for Recurring Instances of Neighboring Genes
TSS: Transcription start site
UK: United Kingdom
