# Supplementary material for: Genome wide association studies are enriched for interacting genes
Source: Res Sq. 2024 Oct 22:rs.3.rs-5189487. Preprint. [Version 2] doi: 10.21203/rs.3.rs-5189487/v2 (PMC11537335; doi:10.21203/rs.3.rs-5189487/v2)
Supplement: Supplement 1 [file NIHPPRS5189487V2-supplement-1.pdf]

Supplementary Files

This is a list of supplementary files associated with this preprint. Click to download.

- SuppInheritedRiskGA.pdf
